# Supplementary material for: Activity- and Enrichment-Based Metaproteomics Insights into Active Urease from the Rumen Microbiota of Cattle
Source: Int J Mol Sci. 2022 Jan 13;23(2):817. doi: 10.3390/ijms23020817 (PMC8776097; doi:10.3390/ijms23020817)
Supplement: Supplementary file 1 [file ijms-23-00817-s001.zip › ijms-1514253-supplementary.pdf]

Supplemental material

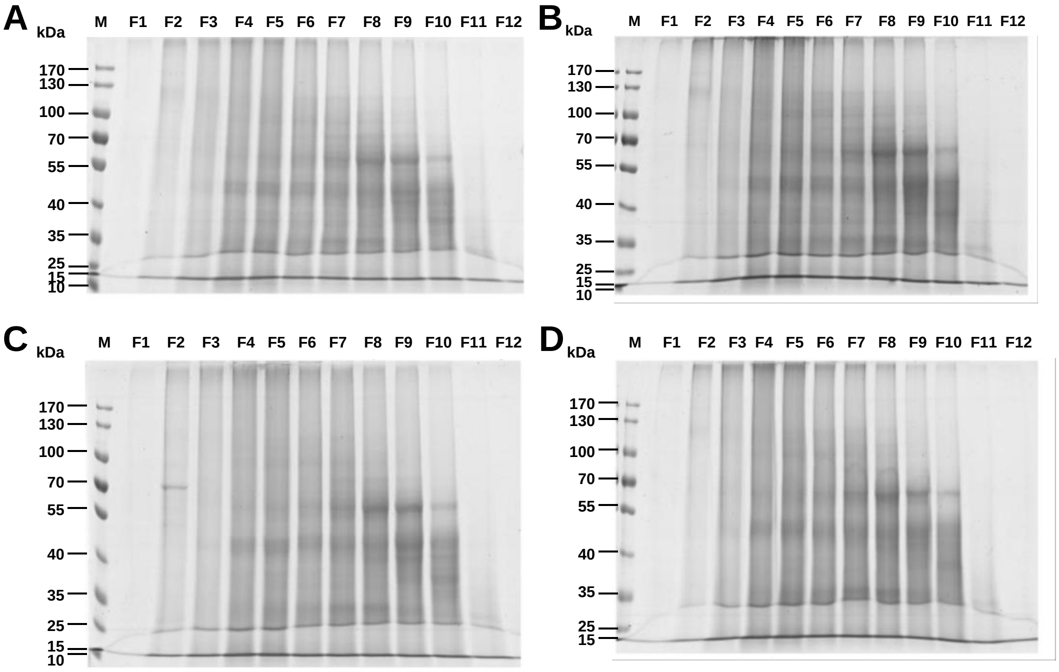

Figure S1. SDS-PAGE gels of purified 12 fractions (F1-F12) from sample 1 (A), sample 2 (B), sample 3 (C), and sample 4 (D).

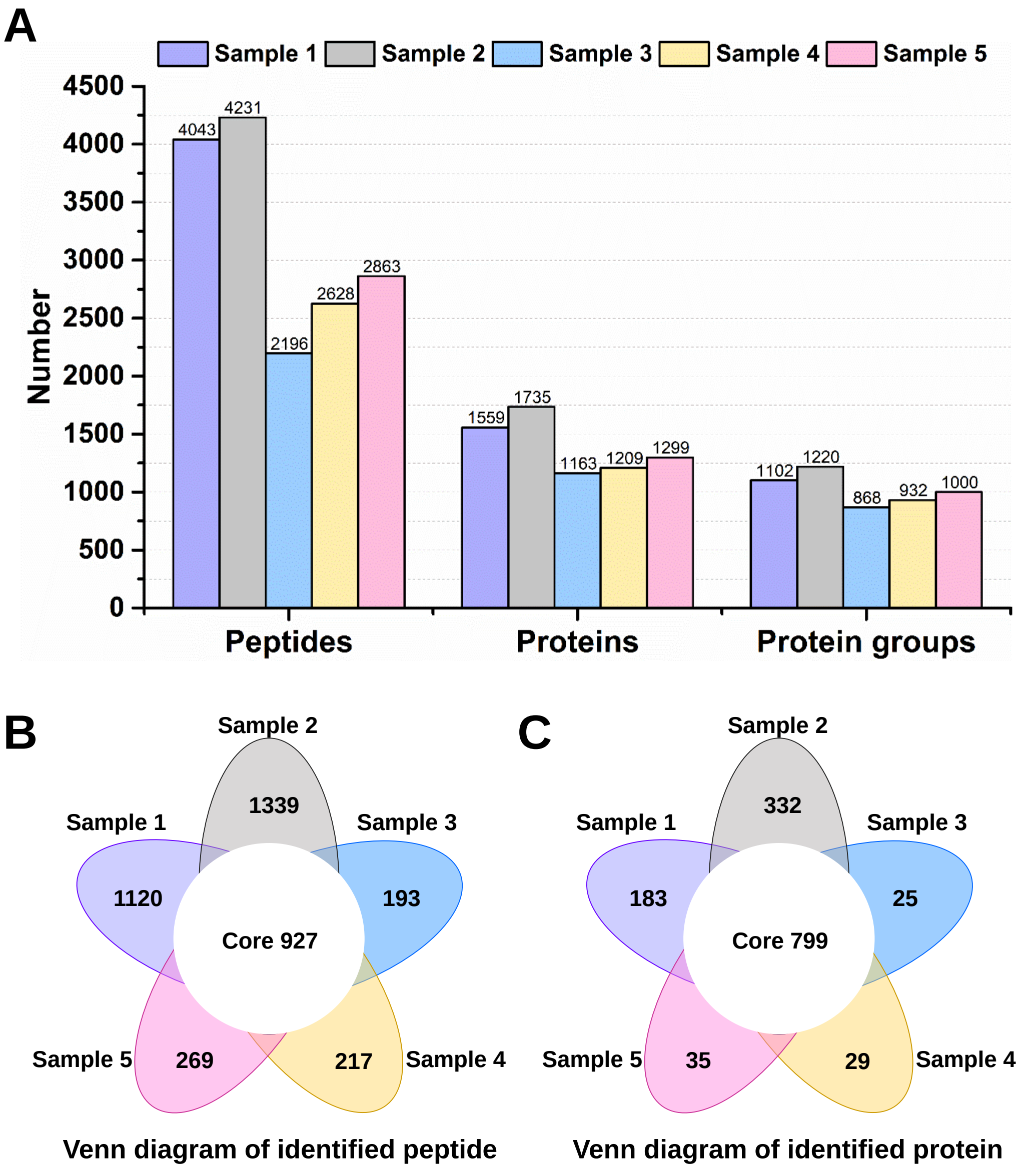

Figure S2. Metaproteomic analyses of fraction 9 with the highest urease activity. (A) Metaproteomic identification of 5 samples. (B) Venn diagram of identified peptides. (C) Venn diagram of identified proteins.
